# Diversity and metabolic potential of the microbiota associated with a soil arthropod

**DOI:** 10.1038/s41598-018-20967-0

**Published:** 2018-02-06

**Authors:** Simon Bahrndorff, Nadieh de Jonge, Jacob Kjerulf Hansen, Jannik Mørk Skovgaard Lauritzen, Lasse Holt Spanggaard, Mathias Hamann Sørensen, Morten Yde, Jeppe Lund Nielsen

**Affiliations:** Department of Chemistry and Bioscience, Aalborg University, Aalborg East, Denmark

## Abstract

Springtails are important members of the soil fauna and play a key role in plant litter decomposition, for example through stimulation of the microbial activity. However, their interaction with soil microorganisms remains poorly understood and it is unclear which microorganisms are associated to the springtail (endo) microbiota. Therefore, we assessed the structure of the microbiota of the springtail *Orchesella cincta* (L.) using 16S rRNA gene amplicon sequencing. Individuals were sampled across sites in the field and the microbiota and in particular the endomicrobiota were investigated. The microbiota was dominated by the families of *Rickettsiaceae*, *Enterobacteriaceae* and *Comamonadaceae* and at the genus level the most abundant genera included *Rickettsia*, *Chryseobacterium*, *Pseudomonas*, and *Stenotrophomonas*. Microbial communities were distinct for the interior of the springtails for measures of community diversity and exhibited structure according to collection sites. Functional analysis of the springtail bacterial community suggests that abundant members of the microbiota may be associated with metabolism including decomposition processes. Together these results add to the understanding of the microbiota of springtails and interaction with soil microorganisms including their putative functional roles.

## Introduction

Decomposition processes in soil ecosystems are strongly influenced by microbes and the soil fauna. The soil fauna play a direct role by feeding on organic material and indirectly stimulating microbial activity through e.g. litter fragmentation, dissemination of microorganisms and grazing activity on fungi and bacteria^1–3^. The soil fauna are thus of ecological importance in the global carbon cycle^4^.

Many soil inhabiting species are colonised by diverse microbial communities playing a pivotal role for their biology. The microbiota in the intestinal tract of soil organisms have received much attention previously (for review see^4,5^) and results show that soil invertebrates contain a rich microbiota with putative symbionts^6–8^. Symbiotic microorganisms can be beneficial to the hosts in many ways, including dietary supplementation, host immune system, and social interactions^4^. The soil fauna can modify the microbial community composition and biomass of the soil^9,10^. It has been suggested that passage through the intestines of the earthworm gut can modify the microbial community composition and thereby the functional diversity of microorganisms in soils inhabited by earthworms^11^. These results suggest a close link between the soil fauna and their associated microbiota, and that some species are dependent on their relationship with the surrounding microorganisms.

Springtails are among the most abundant soil-dwelling arthropods and depending on the habitat can reach densities ranging from 10^2^ to 10^5^ individuals per square meter^3^. Springtails are considered as food generalists and may feed on a great variety of resources, such as fungi, bacteria, mosses, spores, decaying plants, and organic debris^12–14^. Studies using stable isotope analysis have suggested that trophic niches of springtails species have differentiated much and that this has contributed to the species diversity of springtails^15^. Several studies have highlighted the role of springtails as detritivores and they have been shown to stimulate microbial decomposition through soil mixing and indirect catalytic effects on energy flow and nutrient turnover^1,2^. Species belonging to the genera of *Entomobrya*, *Folsomia*, *Orchesella*, and *Tomecerus* have been classified as primary decomposers, feeding on litter material and the adhering fungi and bacteria^15^.

Traditional cultivation studies have previously recovered a number of bacteria from the springtail *Folsomia* *candida* including members of the phyla Proteobacteria (Alphaproteobacteria and Gammaproteobacteria) and Firmicutes^16,17^. Furthermore, it has been shown that some of these bacteria, tentatively identified as *Stenotrophomonas* (formerly *Xanthomonas*) *maltophilia* (Gammaproteobacteria) and *Curtobacterium* sp. (Actinobacteria) were capable of degrading chitin, thus suggesting a functional role for springtails^18^. In other springtail species, a number of Proteobacteria representatives have been detected^19,20^. Further, a recent study applied high throughput Amplicon sequencing to investigate the microbiota of *F*. *candida* using a suppression of the amplification of DNA from the dominant endosymbiont *Wolbachia*^8^. Results showed that applying a suppression treatment was effective against *Wolbachia* and did not interfere with the detection of the most abundant OTUs, although the overall community composition was affected. The most abundantly detected bacterial families included *Bacillaceae* and *Pseudomonadaceae*.

In the present study, the microbiota and endomicrobiota associated with the soil arthropod *O*. *cincta* were characterised. The microbiota of *O*. *cincta* was evaluated from field obtained individuals. Furthermore, in order to assess the degree of intraspecific variability in the composition of bacterial communities, four populations were surveyed across sites on a local scale. Few studies have addressed the variation in the microbiota found within and between populations or locations under natural conditions of invertebrates and for soil animals in particular^21^. Finally, the metabolic potential of the microbiota was explored by assessment of pathways related to various metabolism encoded by these microorganisms.

## Materials and Methods

### Study organism and experimental design

The study was conducted on the springtail *Orchesella cincta* L. (Hexapoda: Collembola). In order to address the microbiota of springtails we collected individuals of *O*. *cincta* directly from the field. Individuals collected in the field were transported back to the laboratory in 2.5 L plastic containers containing a substrate of water-saturated plaster-of-Paris:charcoal mix (ratio 9:1) and twigs until return to the laboratory on the day of collection.

In order to assess the degree of intraspecific variability in the composition of the microbiota, four populations of *O*. *cincta* were collected at the following sites: 1) Sohngårdsholmsparken (57.028194 N 9.948750E) (decimal degrees), 2) Sohngårdsholmsparken (57.026444 N 9.948972E, 3) Højvang Øst, (57.014222 N 9.954861E), and 4) Højvang Øst (57.014389 N 9.954972E). The samples were collected on the 17^th^ of September, 2016. The distances between sampling sites was 195 meters between the two sites at Sohngårdsholmsparken and 20 meters at the two sites at Højvang Øst. The distance between location 1, 2 and 3, 4 was 1.6 km. The locations were similar in terms of soil type and vegetation, which primarily was dominated by scotch pine with sporadic bushes of hawthorn. A minimum of 150 individuals were collected at each site and upon return to the laboratory species identity of specimen was assured following^22^. Subsequently individuals for each site were divided into replicates of 25 individuals. The microbiota and endomicrobiota of three replicates of 25 individuals each were analysed for each site. In order to identify the endomicrobiota, springtails were washed as previously described^23^ with the following modifications: Individuals were washed twice for two minutes in 2.5% (wt/vol) bleach solution (NaClO) and subsequently rinsed twice for two minutes in sterile water. Individuals were subsequently air dried at room temperature. A second group of individuals did not receive any washing treatment and thus included both the endomicrobiota and the ectomicrobiota. It was not possible to assess the age of the field obtained individuals. All samples were stored at −18 ^◦^C until further processing.

### DNA extraction and 16S rRNA gene amplicon sequencing

Total DNA of springtails was extracted from the replicate pools of 25 individuals using the DNeasy® Blood & Tissue Kit (Qiagen, Hilden, Germany) and following the Qiagen supplementary protocol for purification of total DNA from insects with the following modifications: the frozen springtail samples were transferred directly into 200 μL PBS buffer and 180 μL ATL buffer and ground using a disposable microtube Pellet Pestle^©^ (Kimble Chase). Subsequently 20 μL proteinase K (600 mAU/mL) was added and the samples were incubated for 16 h at 56 ^◦^C. DNA from each sample was eluted into 100 µL of AE-buffer. DNA quantity and quality were verified using a Qubit 2.0 fluorometer (Thermo Fisher Scientific, USA) with Qubit dsDNA BR Assay kit (Thermo Fischer Scientific, USA) and by using the TapeStation 2200 with Genomic DNA ScreenTapes (Agilent, USA).

Amplicons were generated through PCR using 10 ng of genomic template DNA per 25 μL reaction (400 nM of each dNTP, 1.5 mM MgSO_4_, 2mU Platinum Taq DNA polymerase HF, 1 × Platinum High Fidelity buffer (Thermo Fisher Scientific, USA) and 400 nM of each primer). The V4 region of the bacterial 16S rRNA gene was amplified using the V4 primer set 515 F GTGCCAGCMGCCGCGGTAA and 806 R GGACTACHVGGGTWTCTAAT^24^. Thermocycler settings were as follows: initial denaturation for 2 min at 95 ^◦^C, followed by 35 amplification cycles (45 s at 95 ^◦^C, 60 s at 50 ^◦^C and 90 s at 72 ^◦^C) and a final extension of 5 min at 72 ^◦^C. Amplicon PCR reactions were run in duplicates and pooled. The amplicons were then purified using AMPure XP bead protocol (Beckmann Coulter, USA) with the following modifications: the sample/bead solution ratio was 5/4, and the purified DNA was eluted in 23 μL of nuclease free water. The generated amplicons were barcoded for sequencing in accordance with the Nextera XT DNA library preparation protocol (Illumina, USA). Library concentration was measured with Quant-iT dsDNA HS Assay kit (Thermo Fisher Scientific, USA) and quality evaluated using D1000 ScreenTapes (Agilent, USA). The samples were sequenced in equimolar concentrations on a MiSeq platform (Illumina, USA) using MiSeq reagent kit v3 (2 × 300 PE).

### Bioinformatic processing and statistical analysis

All sequenced sample libraries were subsampled to 50,000 raw reads. Generated raw reads were quality checked using trimmomatic (v0.32)^25^. Reads were merged using FLASH (v1.2.7)^26^ and subsequently formatted for use with the UPARSE workflow^27^. USEARCH7 was used to dereplicate reads, screen for Phi-X contamination, remove chimeric sequences and cluster into Operational Taxonomic Units (OTUs) at 97% sequence similarity. Taxonomy was assigned using RDP classifier as implemented in QIIME and using SILVA (version 128) as the reference database^28^.

The statistical analyses and visualizations were performed in R version 3.3.3^29^ via RStudio version 1.0.143 (http://www.rstudio.com) using the R packages ampvis^30^ and phyloseq^31^. Biodiversity was explored through the observed number of OTUs and diversity indices ChaoI, and Shannon-Weaver^32,33^. Diversity indices did not meet the assumptions of equal variances, and differences between (endo) microbiota and between locations were thus assessed using the nonparametric Kruskal–Wallis test. Beta diversity was calculated for the microbiota using Bray-Curtis dissimilarity^34^ and unweighted UniFrac metrics^35^. Principal coordinate analysis (PCoA) was used to visualize differences between microbial communities. Only OTUs with an abundant presence (>0.1% of total reads in at least 1 sample) were included in the analysis. The microbial community structure was visualised using heatmaps.

We applied PICRUSt (v1.1) (Phylogenetic Investigation of Communities by Reconstruction of Unobserved States) in order to identify how phylogenetic differences of the microbiota of springtails impact the microbial metabolic potential in accordance with the recommended workflow^36^. PICRUSt uses an extended ancestral-state reconstruction algorithm to predict which gene families are present and then combines gene families to estimate the composite metagenome. This allows to predict the functional composition of the metagenome using marker gene data (such as 16S rRNA) and a database of reference genomes. Data was analysed using STAMP software^37^ and R. Metabolic cycles and pathways of importance to the soil ecosystem were selected from KEGG^38^ and examined in detail. A heatmap of the relative abundances of the 15 most abundant OTUs was generated based on the individual OTUs contributions to the selected pathways. The heatmap was ordered with help of a dendrogram based on unweighted UniFrac distances between samples.

All amplicon data are available at European Nucleotide Archive (ENA) under project number PRJEB21365 (http://www.ebi.ac.uk/ena/data/view/ PRJEB21365).

## Results

### Bacterial diversity associated with a soil arthropod

The 16S rRNA gene amplicon sequencing yielded a total of 1,091,167 reads with an average number of 45,465 ± 8,205 reads per sample (mean ± SD). A total number of 1,681 unique OTUs were identified and with an average number of 335 ± 190 OTUs per sample. Rarefaction curves of generated OTUs approximated a horizontal line indicating that the majority of microbial diversity was captured (Supplementary Figure 1). Based on this, a minimum number of 8,000 sequences per sample were considered suitable for analysis.

**Figure 1:**
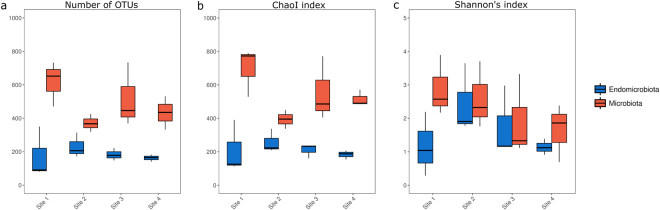
Alpha diversity measurements of the microbiota and endomicrobiota of *Orchesella cincta* sampled across locations. Boxplot displaying the observed number of OTUs (**a**), the estimated richness (**b**); ChaoI index) and evenness (**c**); Shannon’s index) (n = 3). The boxplot bounds the interquartile range (IQR) divided by the median, the whiskers extend to 1.5 × IQR beyond the box.

The number of OTUs observed and the estimated richness (Chao1 index) for the microbiota was significantly higher compared to the endomicrobiota (p < 0.001), but this was not the case for evenness (Shannon’s index) (p = 0.119) (Fig. 1). When comparing across sites, the observed number of OTUs, the Chao1 index and Shannon’s index differed between sites although not significantly (p > 0.05).

### Composition of microbiota associated with a soil arthropod

**Figure 2:**
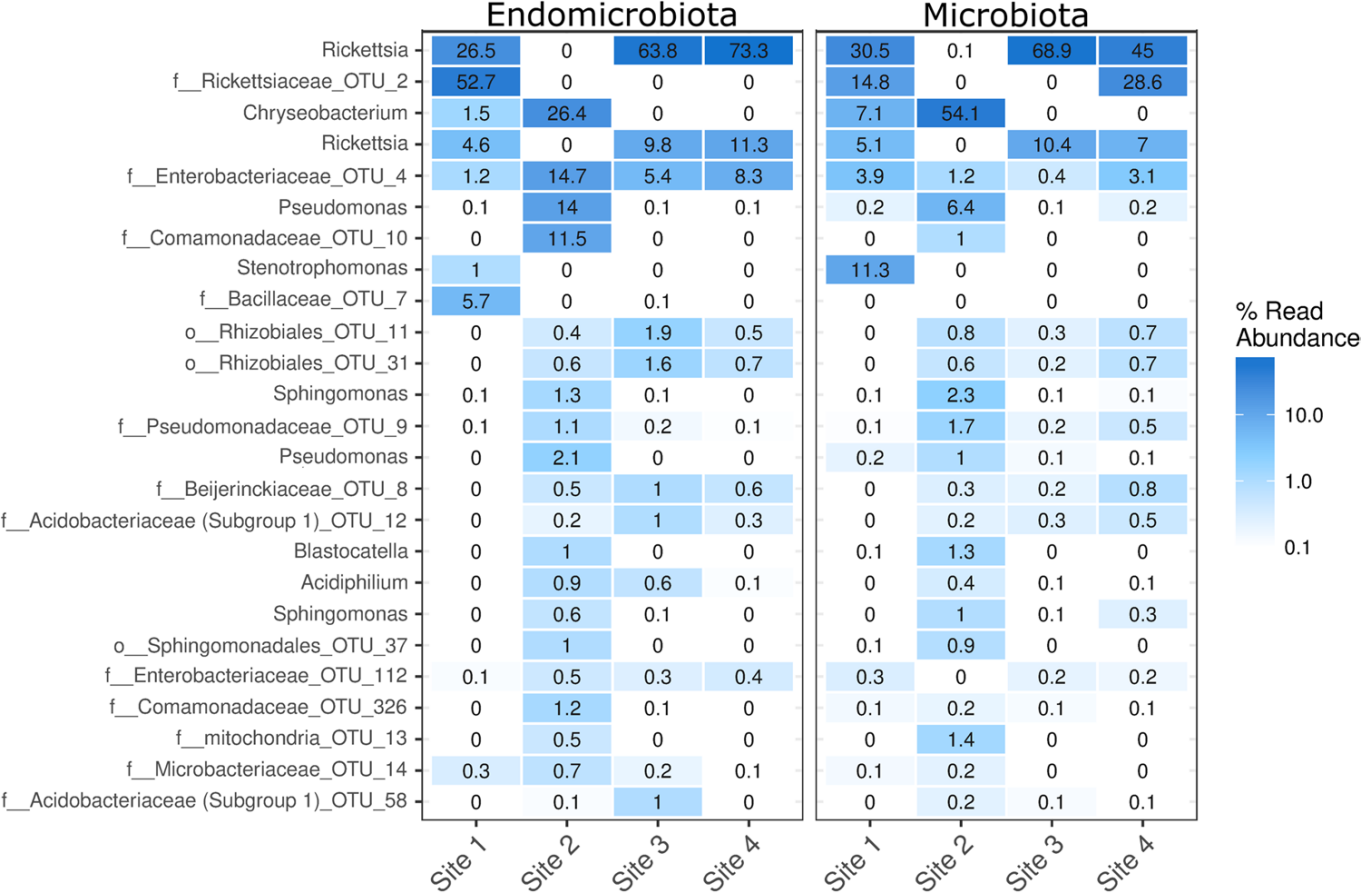
Composition and abundance of the springtail microbiota. Heatmap representing the 25 most abundant OTUs detected across sites, with the genus level or highest possible taxonomic classification displayed. Samples are sorted by microbiota and endomicrobiota and site.

The dominant bacterial taxa associated with *O*. *cincta* differed for the microbiota and endomicrobiota and across sites (Fig. 2). The most abundant OTUs contained representatives of the genera *Rickettsia*, *Chryseobacterium* and *Pseudomonas*, and the family of Rickettsiaceae and Enterobacteriaceae. The microbiota at site 1, 3 and 4 were dominated by *Rickettsia* and by *Chryseobacterium* at site 1 and 2. The genus *Pseudomonas* and representatives of the family *Enterobacteriaceae* were present across all sites, but the latter was less abundant. The endomicrobiota showed similar trends in microbial community structure, but minor shifts in abundance were observed for the most abundant OTUs, including the genus *Chryseobacterium* at site 1 and 2 and *Rickettsia* especially at site 4. OTUs representing *Pseudomonas* and *Comamonadaceae* were also more prevalent in the endomicrobiota. Furthermore, an OTU representing *Rickettsiaceae* which was abundant at site 4 in the microbiota was not detected in the endomicrobiota.

**Figure 3:**
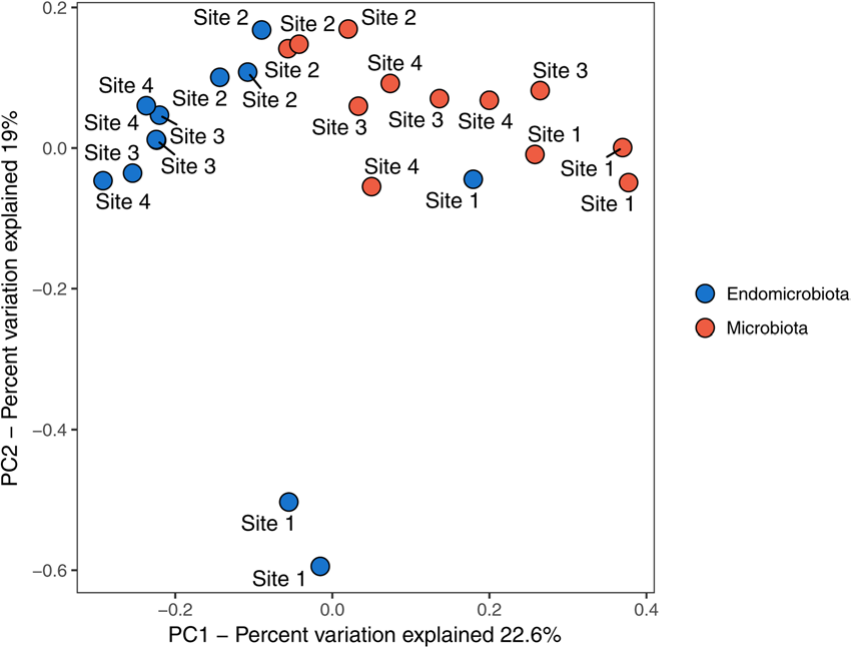
Beta diversity analysis of the microbiota of *Orchesella cincta*. Principal coordinate analysis (PCoA) on unweighted UniFrac distances between samples. Samples are colored by microbiota and endomicrobiota.

Ordination using unweighted UniFrac distances showed that the microbial community of the endomicrobiota diverged from the microbiota and separated into clusters with the exception of one sample from site 1 (Fig. 3). The first ordinate (PCoA1) explains 22.5% of the variation and separates the microbiota and the endomicrobiota, whereas the second ordinate (PCoA2) explains 18.9%. A significant effect of site was shown using MANOVA on a Bray-Curtis dissimilarity matrix generated between samples for the microbiota (adonis test, p < 0.001, R^2^ = 0.5296), but not between the microbiota and endomicrobiota (adonis test, p < 0.711, R^2^ = 0.0249).

### Metabolic potential of soil arthropod associated microbiota

**Figure 4:**
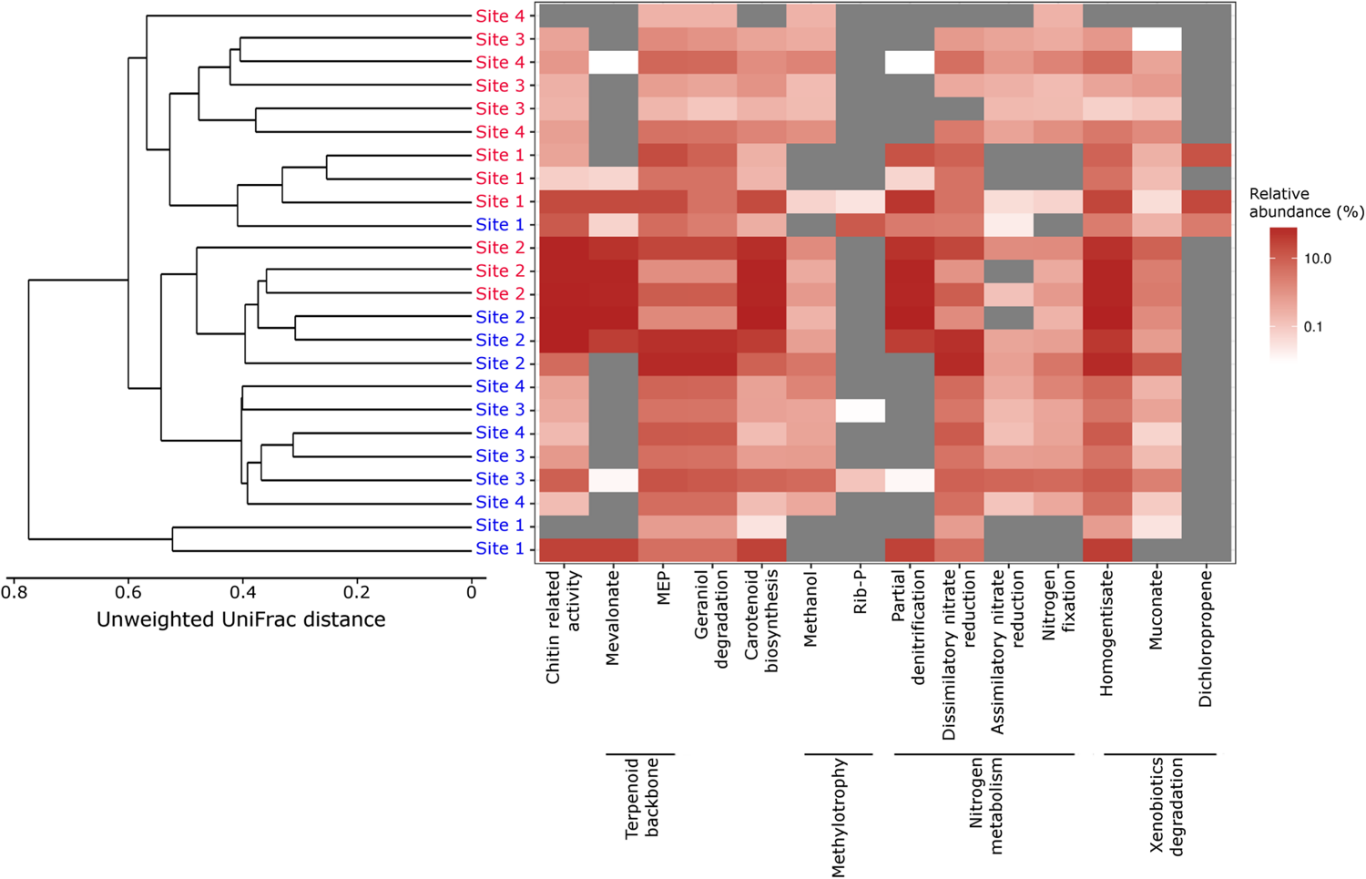
Predicted metabolic profiles. Heatmap of the association of the 15 most abundant OTUs of the gut flora of the springtail *Orchesella cincta* with Level 2 KEGG pathways. MEP = methylerythritol phosphate pathway, Rib-P = Ribosome P. Heatmap is sorted using a dendrogram based on unweighted UniFrac distances between samples. Samples are colored by microbiota (red) and endomicrobiota (blue).

Metabolic potential predictions of the microbiota as determined by selected level 2 KEGG pathways are shown in Fig. 4 and supplementary Table 1. Assessment of the obtained metabolic profiles for the 15 most abundant members of the microbiota revealed a prominent and organism-specific presence of genes involved in terpenoid metabolism through two different distinct pathways (mevalonate and methylerythritol phosphate pathway). Furthermore, several microorganisms with potential nitrogen metabolisms (dissimilatory and assimilatory nitrate reduction, denitrification and nitrogen fixation) were also observed and predominantly present in the endomicrobiota, suggesting potentially activity or uptake of microorganisms with these activities in the gut flora (Fig. 4). Enrichments of microorganisms associated with potential activities within the KEGG classifications: metabolism and xenobiotics biodegradation, included the transformation of homogentisate and muconate. Furthermore, genes involved in carotenoid biosynthesis and chitin related pathways were also observed abundantly. A dendrogram based on unweighted UniFrac distances between samples of 16S rRNA gene sequences of the 15 most abundant members showed that most OTUs of the microbiota or endomicrobiota grouped together. Similarly, there was some structure according to site of collection, where individuals from site 1 or 2 grouped (Fig. 4).

## Discussion

The aim of the present study was to characterise the diversity and potential functional role of the microbiota associated with the soil arthropod *O*. *cincta* across collection sites. We analysed the microbial diversity and in particular the interior (endomicrobiota) of individuals sampled across sites. Although soil inhabiting species are colonised by diverse microbial communities playing a pivotal role for their biology, the interaction between soil animals and their microorganisms are still poorly understood including their functional role^4^. We found that the microbial communities were distinct for the interior of the springtails for measures of population diversity and the microbiota exhibited structure according to collection sites.

### Microbiota richness and differences across treatments

Alpha diversity measurements in the present study were comparable with other studies investigating the microbiota of arthropods^23,39–42^, where the observed number of OTUs ranged from 81 to 734 across sites and Shannon’s diversity index from 3.4 to 4. Furthermore, there were significant differences for alpha and beta diversity measurements in the endomicrobiota and microbiota, and between sites. Significant differences in observed number of OTUs and in the estimated richness (Chao1 index) suggest that differences between the endomicrobiota and the microbiota are mainly driven by rare OTUs, which is supported by the UniFrac analysis. These results are in accordance with other studies addressing the role of the ecto- and endomicrobiota of arthropods^43^.

Individuals were collected from locations in close proximity of each other. However, studies have shown large differences in the microbiota of insects both within and between populations^21^. In the present study, we found a significant effect of site on beta diversity of the microbiota, which was evident, both when using unweighted UniFrac distances and Bray-Curtis dissimilarities. This is in agreement with other studies^8,20^ and even though population effects were small these results suggest some environmental selection.

### Taxonomic composition of the springtails microbiota

In the present study, we were able to identify multiple populations of bacteria associated to the microbiota of *O*. *cincta*. In particular, the genera *Rickettsia*, *Chryseobacterium*, and *Pseudomonas* were abundant and present in both the microbiota and endomicrobiota, but with differences across sites. Similar dominance of *Rickettsia* and *Pseudomonas* has been established in another springtail, *Folsomia candida*^20^, but not *Chryseobacterium*. Members of the genus *Rickettsia* are intracellular symbionts of eukaryotes, but most strains are vertically inherited symbionts of invertebrates and it has been shown that *Rickettsia* symbionts in insects can have diverse effects ranging from influencing host fitness to manipulating reproduction^44,45^. In the springtail *Onychiurus sinensis*, *Rickettsia* has been found in the male and female gonads^46^. A number of microorganisms were present in both the microbiota and endomicrobiota and across most sites, but were all found at lower abundances. These included genera such as *Sphingomonas* and *Acidiphilium* and OTUs representing the families *Pseudomonadaceae*, *Beijerinckiaceae*, and *Acidobacteriaceae*. Except for *Pseudomonadaceae*, these groups of bacteria have not been reported in springtails before. Representatives of OTUs belonging to the family *Enterobacteriaceae* and genus *Pseudomonas* were present across all sites and in both the microbiota and endomicrobiota. Several of these bacterial populations have been found in different insect species and they have been suggested to have beneficial roles to the host. For example, the microbiota of the mountain pine beetle is dominated by members of the genus *Pseudomonas*, which has been shown to possess a majority of the genes involved in terpene metabolism^47^. This may also be of relevance to plant litter decomposition by springtails. *Stenotrophomonas* was only detected at one site, but has also been reported by other studies^20^. Members of this genus has been associated with nitrogen fixation and cellulolytic activity in insect species^48,49^.

### Metabolic profiles associated to the springtail microbiota

PICRUSt was applied to the 16S rRNA gene amplicon data as a metagenome inference method. The metabolic potential of the springtail microbial community of the present study revealed abundant representation of genes representing pathways related to metabolism including nitrogen metabolism and degradation of xenobiotics, chitin related activity, terpenoid metabolism and carotenoid biosynthesis. These enrichments of metabolic pathways may be due to the diet of *O*. *cincta*. Epedaphic species of springtails such as *O*. *cincta* are often found on and in trees and with a large part of their gut filled with plant material^50^ including epiphytic algae like *Desmococcus*^13^. Plant material is generally rich in cellulose, hemicellulose, and soluble carbohydrates, but also poor inassimilable nitrogen^51^, although algae like *Desmococcus* can be considered n-rich food (4.3%)^52^. Co-evolution of beneficial microorganisms with the host can fundamentally shape animal physiology and behaviour and the microbiota may modulate the availability of ingested nutrients and thus also the energy available to the host. Based on their diet, it is likely that *O*. *cincta* requires symbiotic relationships with microorganisms that supply assimilable carbon and nitrogen sources as observed in other insect species^48,53^.

In accordance with this, we found representation of genes involved in nitrogen metabolism (nitrogen fixation, partial denitrification, assimilatory and dissimilatory nitrate reduction) in most of the abundant microorganisms, suggesting that symbionts may provide springtails with nitrogen compounds or help recycling nitrogen waste of the host^53,54^. We also find evidence for enrichment of pathways related to muconate lactonizing enzymes that convert cis,cis-muconates to muconolactones in soil microbes as part of the ß-ketoadipate pathway. This aerobic catabolic pathway converts aromatics such as the breakdown products of lignin, through catechol and protocatechuate to citric acid cycle intermediates^55^. Lignin is a component of cell walls of plants and algae, which is both part of the diet of *O*. *cincta*. Some of these enzymes are also dehalogenate muconate derivatives of xenobiotic haloaromatics^55^. The presence of these bacteria across most sites and samples thus suggest that the gut microflora is part of the breakdown of components of the diet of *O*. *cincta*. There were also presence of genes involved in production of homogentisate and thus involved in metabolism of aromatic amino acids, such as tyrosine and phenylalanine^56^. Both of these amino acids play a vital role for growth in insects and results have shown high levels of tyrosine of the outermost lipid layer of freshly shed cuticles of springtails^57^. Endosymbionts can provide insects with essential amino acids, where Gammaproteobateria has been shown to be involved in the biosynthesis of phenylalanine^58^. It is thus likely that homogentisate related activity could play a role in the metabolism of springtails. Chitin is a major component of fungal cell walls, but also the exoskeleton of springtails and a survey of 18 species of springtails showed the presence of the chitinase activity in 16 of these species^59^. However, the authors were not able to discriminate whether the digestive enzymes, responsible for the breakdown of food items, were produced by the springtail or excreted in the gut by the microflora. The results of the present study suggest that the microflora indeed play a role in the excretion of chitinase and contribute to the breakdown of food items. Similarly, the diet and habitat of *O*. *cincta* can help explain the presence of pathways involved in metabolism of terpenes^60,61^. Carotenoids also serve important biological functions, but animals are generally unable to synthesise these pigments and instead obtain them from food. Many insects may have limited access to carotenoids in their diet, and for some insects endosymbionts can serve as an alternative source of carotenoid biosynthesis^62^. Soil algae have been shown to contain different carotenoids^63^, but their role for springtails remains to be tested.

It is worth noting that the 15 most abundant OTUs across site and treatment were also distributed differently in terms of metabolic potential (gene) abundances between sites and treatments. Thus, the metabolic potential also differs between sites and treatments. For example, site 2 show a higher abundance of OTUs with putative roles in chitin related activity, mevalonate, carotenoid biosynthesis, denitrification and homogentistate. At present, it is unclear what environmental factors that drive such differences. Further studies are needed to clarify if site specific differences in OTUs and metabolic potential thus affect species’ function across environments. PICRUSt predicts functional profiling of microbial communities using 16S rRNA marker gene sequences without deep metagenomic sequencing. The prediction accuracy of PICRUSt has been tested to diverse metagenomic data sets such as humans, soils, other mammalian guts and the Guerrero Negro, showing that the phylogenetic information contained in 16S marker gene sequences is sufficiently well correlated with genomic content to yield accurate predictions when related reference genomes are available^36^. It can thus predict and compare probable functions across a wide range of samples although it is limited by the availability of closely related reference genomes in public databases from similar habitats.

## Conclusions

In the present study, characterisation of the microbial communities associated with springtails showed that the identified microbiota was distinct for the interior of the springtails in terms of diversity and the microbiota exhibited composition according to collection sites. Furthermore, abundant bacterial populations with putative roles in nitrogen metabolism, breakdown of components of the diet and secondary plant metabolites were identified. Functional analysis of the springtail bacterial community supported their proposed role in soil metabolism including decomposition processes and biodegradation. Collectively these results enhance our understanding of the microbiota of springtails and their interaction with soil microorganisms including putative functional roles of the microbiota.

## Electronic supplementary material

Supplementary files
