# Supplementary material for: Diversity and metabolic potential of the microbiota associated with a soil arthropod
Source: Sci Rep. 2018 Feb 6;8:2491. doi: 10.1038/s41598-018-20967-0 (PMC5802828; doi:10.1038/s41598-018-20967-0)
Supplement: Supplementary file 1 — Supplementary files [file 41598_2018_20967_MOESM1_ESM.doc]

**Title**

**Diversity and metabolic potential of the microbiota associated with a soil arthropod**

**Authors**

Simon Bahrndorff ^1^, Nadieh de Jonge ^1^, Jacob Kjerulf Hansen ^1^, Jannik Mørk Skovgaard Lauritzen ^1^, Lasse Holt Spanggaard ^1^, Mathias Hamann Sørensen ^1^, Morten Yde ^1^ and Jeppe Lund Nielsen ^1*^

**Figure S1. Rarefaction curve based on 97% operational taxonomic units (OTUs) sorted by microbiota and endomicrobiota.**


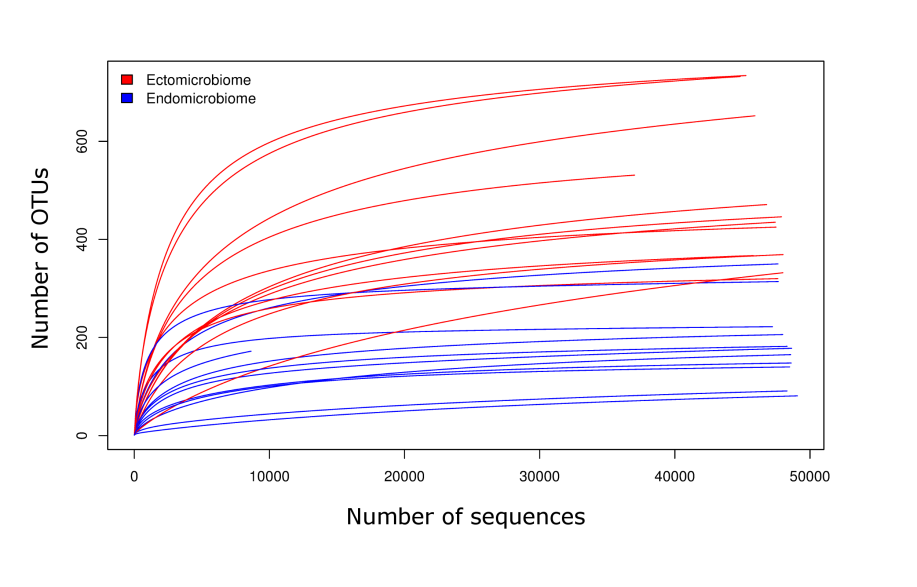


**Table S1. Predicted metabolic profiles (Level 2 KEGG pathways) of the gut flora of the springtail *Orchesella cincta*.**

**+ = 1 gene / partial pathway, ++ = many genes / complete pathway. Meval = mevalonate pathway, MEP = methylerythritol phosphate pathway, Rib-P = Ribosome P.**
